# Supplementary material for: 3D electron-beam writing at sub-15 nm resolution using spider silk as a resist
Source: Nat Commun. 2021 Aug 26;12:5133. doi: 10.1038/s41467-021-25470-1 (PMC8390743; doi:10.1038/s41467-021-25470-1)
Supplement: Supplementary file 1 — Supplementary information [file 41467_2021_25470_MOESM1_ESM.pdf]

Supplementary information

$$\lambda = \frac{h}{\sqrt{2m_0eV}\left(1 + \frac{eV}{2m_0c^2}\right)}$$

λ: de Broglie wavelength (nm)

h: Planck's constant (6.626 x 10^{-34} J s)

V: accelerating voltage (V)

e: electron charge (1.602 x 10^{-19} C)

m_0: electron mass (9.109 x 10^{-28} g)

c: speed of light (2.998 x 10^{8} m s^{-1})

Accelerating voltage （kV）	De Broglie electron wavelength （nm）
1	0.03876
5	0.01726
10	0.01215
25	0.00757
50	0.00536
100	0.00370

Supplementary Figure 1. The de Broglie wavelengths of electrons with different accelerating voltages.

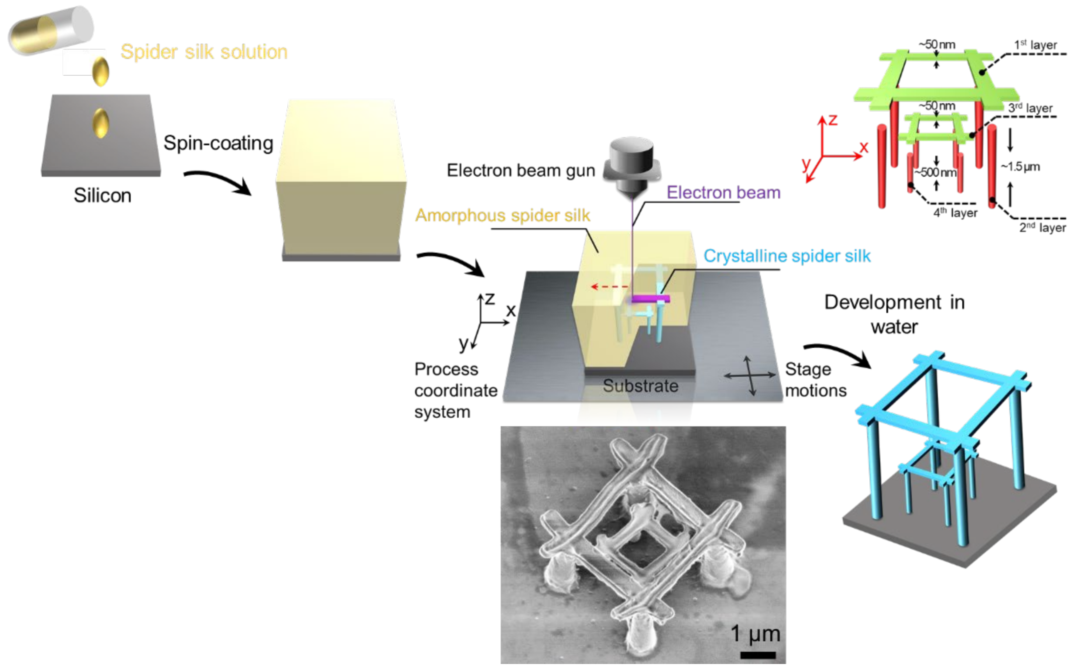

Supplementary Figure 2. Illustration of the 3d EBL process in spider silk.

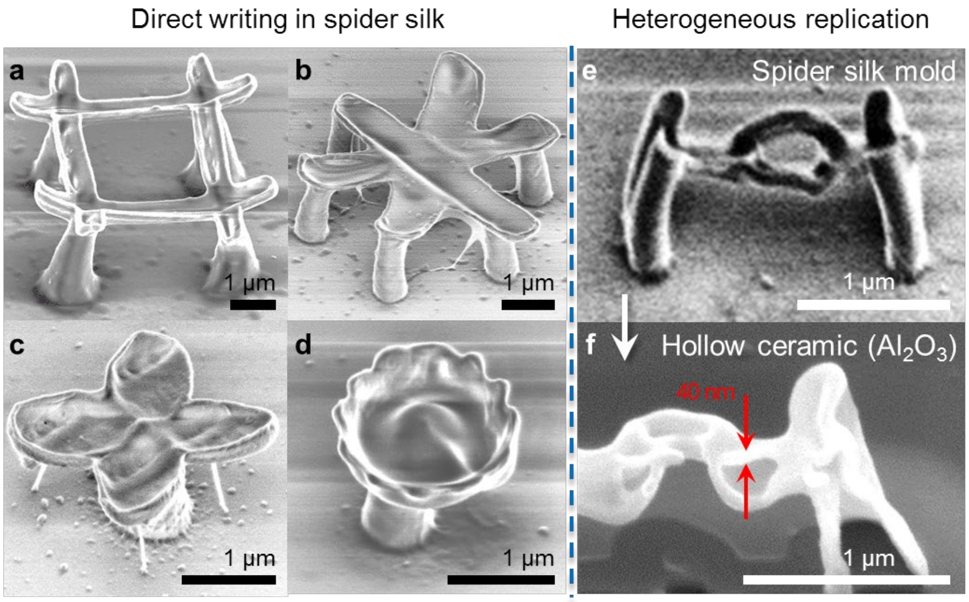

Supplementary Figure 3. Complex 3d nanoarchitechtures fabricated using 3d EBL. a, Nanoscaffold. **b,** Nanoblade. **c,** Nanoclover. **d,** Nanoflower. **e, f,** Solid spider silk template (e) and the corresponding hollow Al_2O_3 nanobeam after the Atomic Layer Deposition (ALD) coating (f).

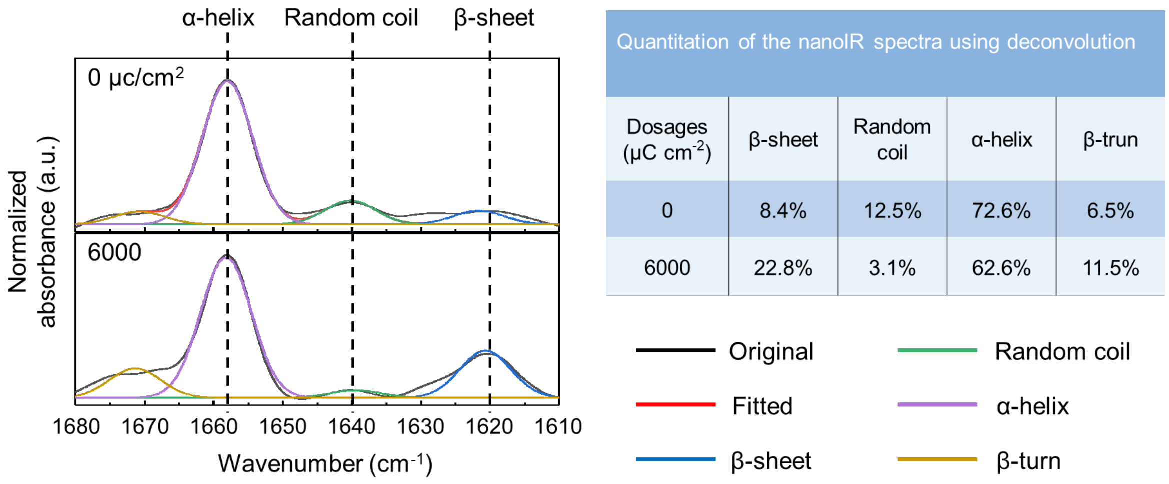

Quantitation of the nanoIR spectra using deconvolution				
Dosages (μC cm^{-2})	β-sheet	Random coil	α-helix	β-trun
0	8.4%	12.5%	72.6%	6.5%
6000	22.8%	3.1%	62.6%	11.5%

Supplementary Figure 4. Nanoscale analysis of conformational transition of spider silk proteins under electron irradiation using AFM-IR (Atomic force microscopy - infrared spectroscopy).

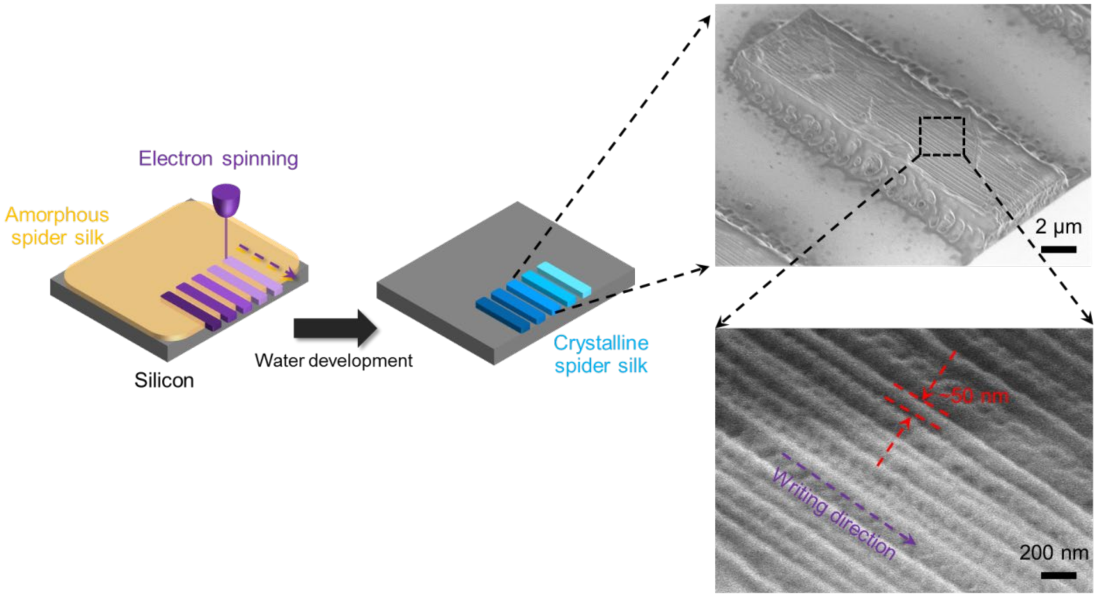

Supplementary Figure 5. Nanofibers of spider silk protein along electron beam writing direction.

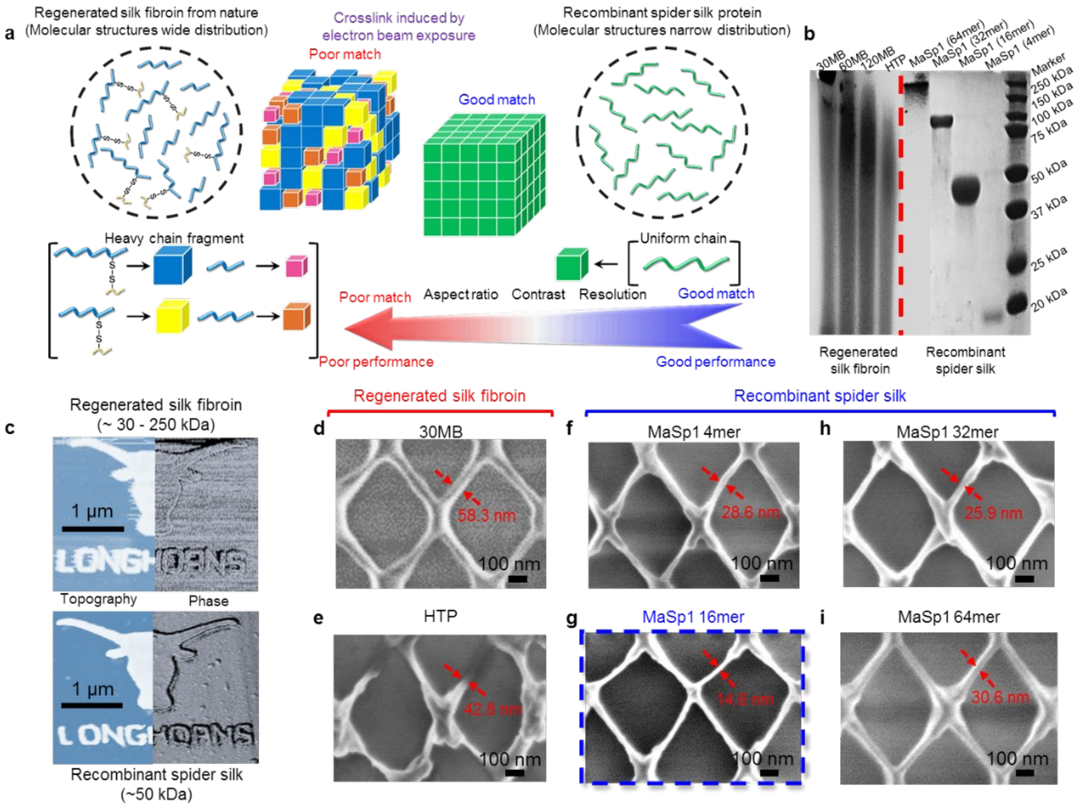

Supplementary Figure 6. High performance of recombinant spider silk protein-based bioresist. **a,** Silk proteins-based nanostructuring using electron beam lithography. **b,** Molecular features of the regenerated silk fibroin (30MB, 60MB, 120MB and HTP) and recombinant spider silk protein (MaSp1 64-mer, 32-mer, 16-mer and 4-mer) analyzed by SDS-PAGE. MB: Minute-boiled. HTP: High temperature and pressure. **c-i,** Higher contrast (c) and resolution (d-i) approaching the molecular level of the recombinant spider silk protein than regenerated silk fibroin.

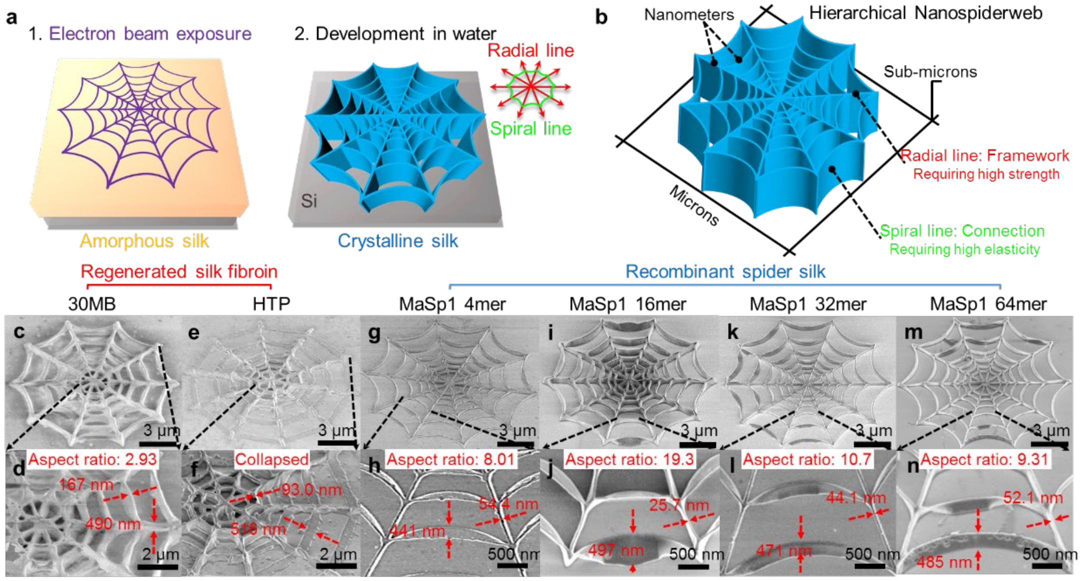

Supplementary Figure 7. Comparative study of the performances of silk fibroin and spider silk proteins for hierarchical nanostructuring. a, Schematics of the nanoweb fabrication process using all-electron direct writing. **b,** Hierarchical nanospiderweb modeling. **c-n**, SEM images of the obtained nanowebs with varying aspect ratios using regenerated silk fibroin and recombinant spider silk.

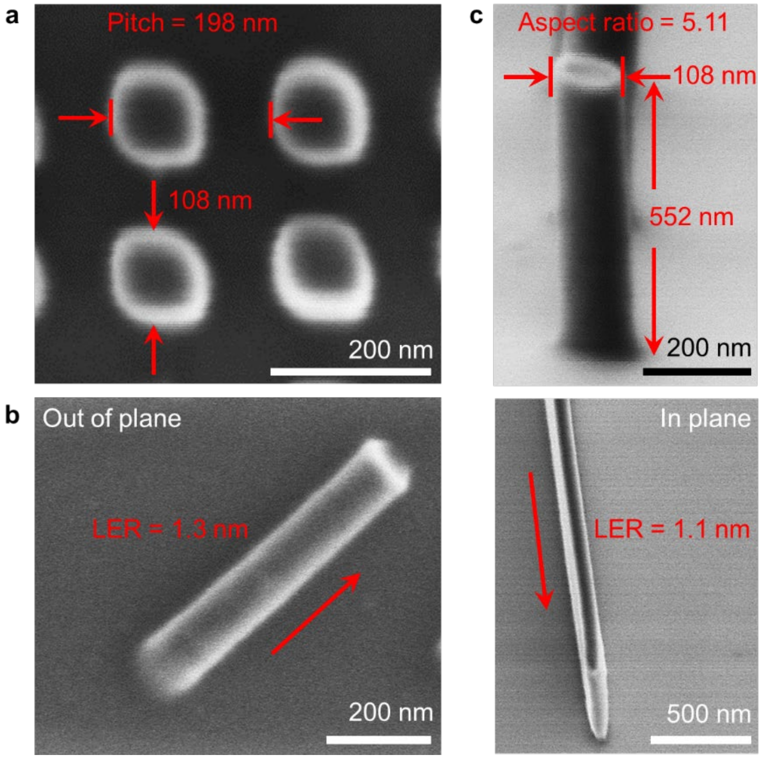

Supplementary Figure 8. The pitch (a), line edge roughness (b) and aspect ratio (c) of the nanostructures fabricated using 3d EBL. LER: line edge roughness.

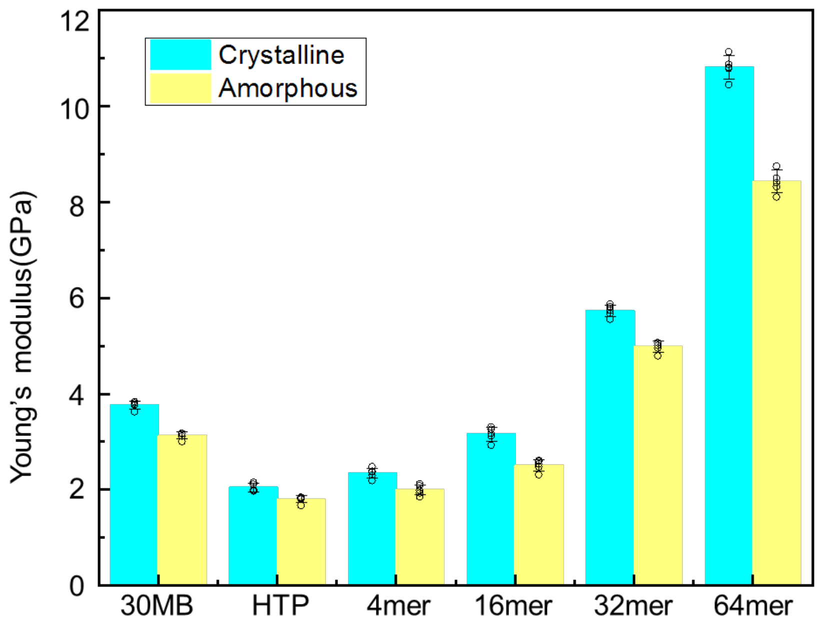

Supplementary Figure 9. Mechanical properties of regenerated silkworm silk protein (30MB and HTP) and recombinant spider silk protein MaSp1 (4-64mer). n = 5 for each group. The error bars denote standard deviations of the mean.

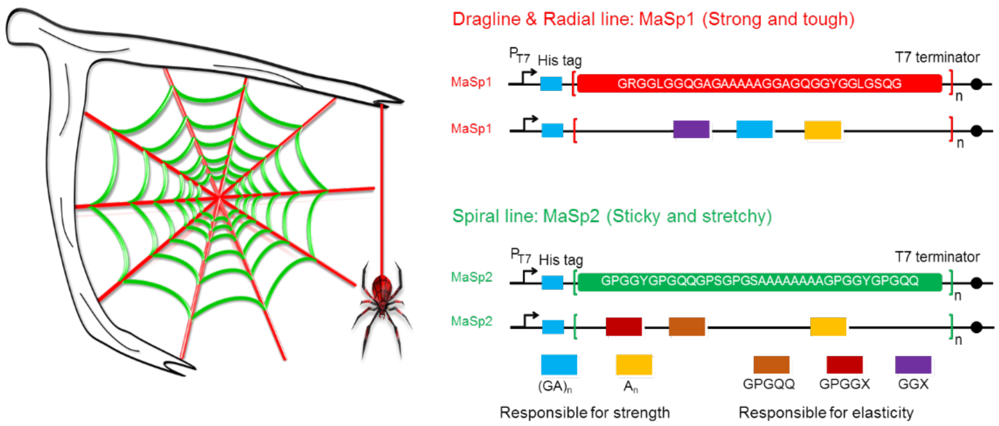

Supplementary Figure 10. Amino acid structures and functions of recombinant spider silk proteins used in 3d EBL.

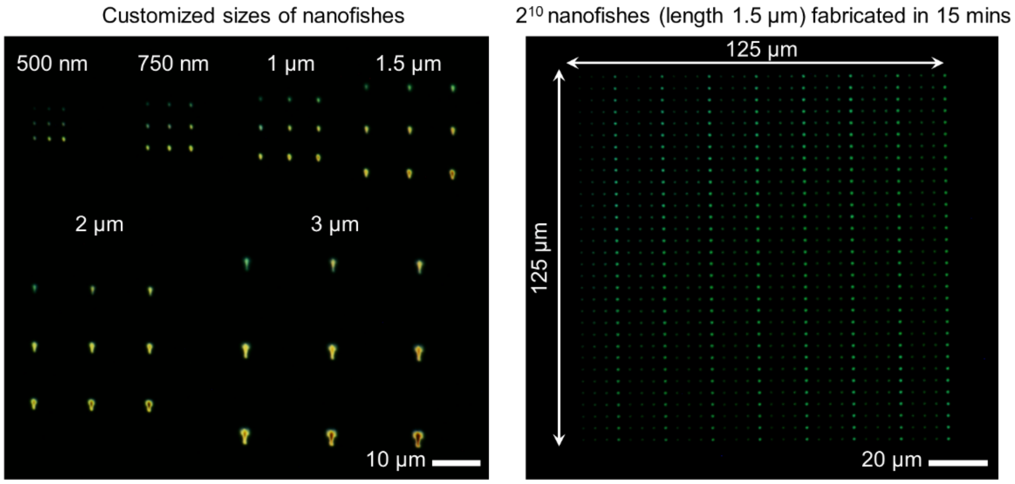

Supplementary Figure 11. Fluorescence microscope photographs of the customized and scalable production of the nanofishes in spider silk.

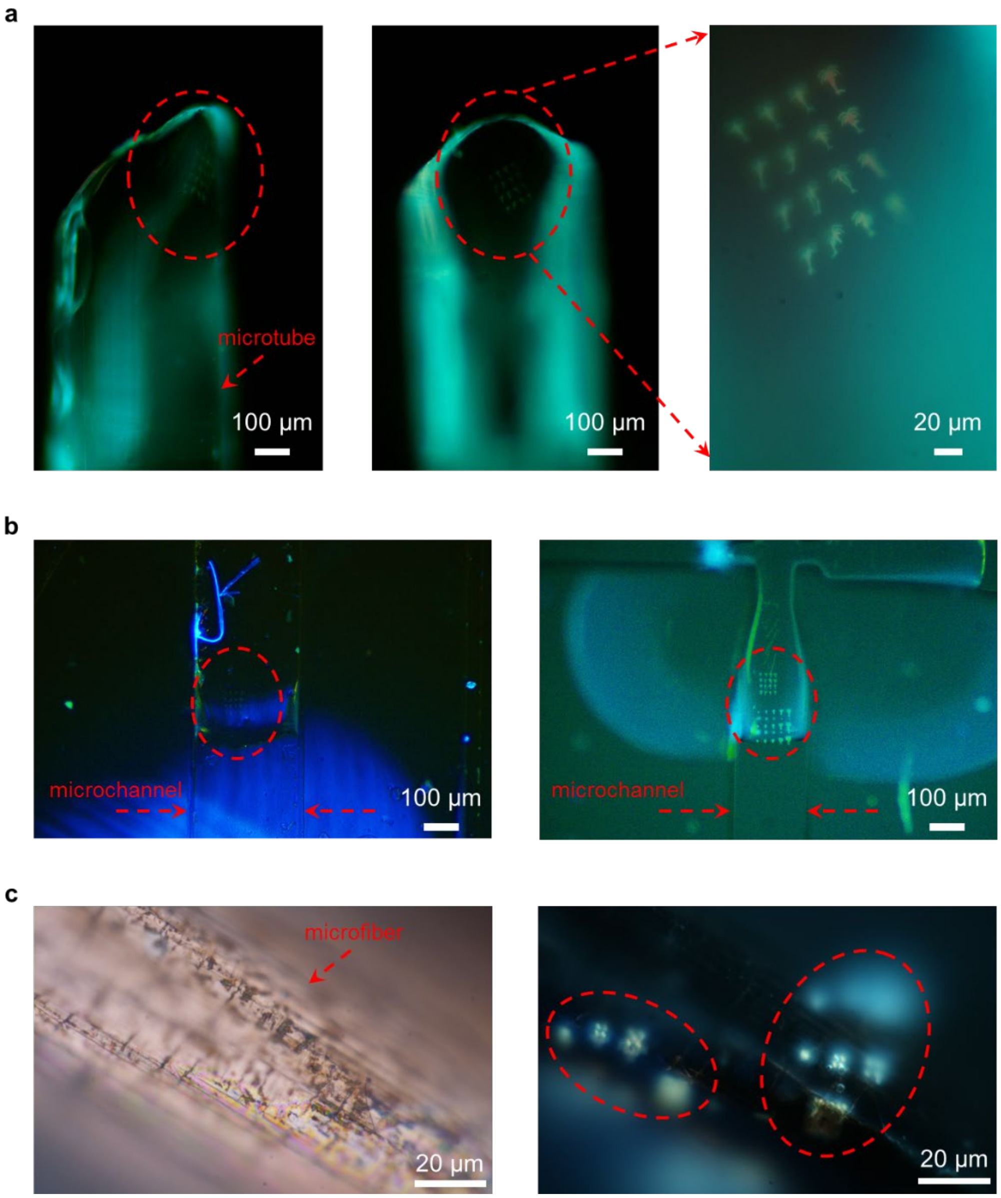

Supplementary Figure 12. Spider silk nanofishes fabricated in the microcapillary tube, microfludic channel and on the curved surface of microfiber. a, b, Fluorescence microscope photographs. **c,** Optical microscope photograph (left) and fluorescence microscope photograph (right).

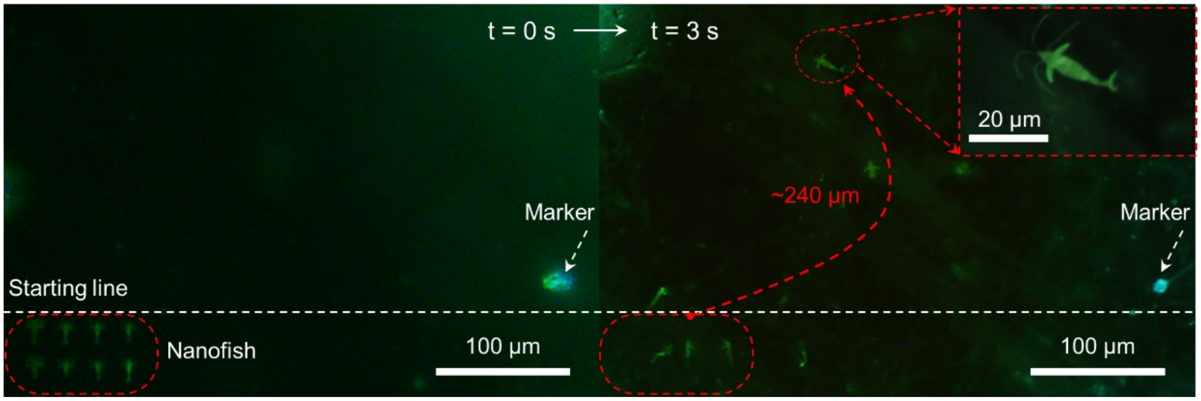

Supplementary Figure 13. The velocity of the spider silk protein nanofish.
