# Supplementary material for: 3D electron-beam writing at sub-15 nm resolution using spider silk as a resist
Source: Nat Commun. 2021 Aug 26;12:5133. doi: 10.1038/s41467-021-25470-1 (PMC8390743; doi:10.1038/s41467-021-25470-1)
Supplement: Supplementary file 3 — Reporting Summary [file 41467_2021_25470_MOESM3_ESM.pdf]

nature portfolio

Corresponding author(s): Tiger H Tao

Last updated by author(s): Jul 20, 2021

Reporting Summary

Nature Portfolio wishes to improve the reproducibility of the work that we publish. This form provides structure for consistency and transparency in reporting. For further information on Nature Portfolio policies, see our Editorial Policies and the Editorial Policy Checklist.

Statistics

For all statistical analyses, confirm that the following items are present in the figure legend, table legend, main text, or Methods section.

n/a	Confirmed	
☐	☒	The exact sample size (*n*) for each experimental group/condition, given as a discrete number and unit of measurement
☐	☒	A statement on whether measurements were taken from distinct samples or whether the same sample was measured repeatedly
☒	☐	The statistical test(s) used AND whether they are one- or two-sided<br>*Only common tests should be described solely by name; describe more complex techniques in the Methods section.*
☒	☐	A description of all covariates tested
☒	☐	A description of any assumptions or corrections, such as tests of normality and adjustment for multiple comparisons
☐	☒	A full description of the statistical parameters including central tendency (e.g. means) or other basic estimates (e.g. regression coefficient) AND variation (e.g. standard deviation) or associated estimates of uncertainty (e.g. confidence intervals)
☒	☐	For null hypothesis testing, the test statistic (e.g. *F*, *t*, *r*) with confidence intervals, effect sizes, degrees of freedom and *P* value noted<br>*Give P values as exact values whenever suitable.*
☐	☒	For Bayesian analysis, information on the choice of priors and Markov chain Monte Carlo settings
☒	☐	For hierarchical and complex designs, identification of the appropriate level for tests and full reporting of outcomes
☒	☐	Estimates of effect sizes (e.g. Cohen's *d*, Pearson's *r*), indicating how they were calculated

Our web collection on statistics for biologists contains articles on many of the points above.

Software and code

Policy information about availability of computer code

Data collection	Scanning electron microscope: Hitachi-4800, Japan<br>Fluorescent imaging: Carl Zeiss A1, Germany<br>Micro-Raman spectrum: Renishaw Raman microscope, UK<br>Nanoscale infrared spectrum: Anasys Instruments NanoIR system, USA<br>Nanoscale mechanical properties test: Multimode VIII scanning probe microscope, Bruker, USA<br>Near infrared imaging: LEICA / VISTEC INM 100, Germany<br>SDS-PAGE: Microtek Bio-5000 Plus scanner
Data analysis	Monte Carlo modeling: Casino v2.51, University of Sherbrooke<br>Fluorescent image analysis: ZEN lite 3.4<br>Nanoscale mechanical properties analysis: Nanoscope Analysis 1.5<br>Band intensity analysis: Image J 1.52u<br>Band decomposition analysis: OPUS software package (version 4.2)<br>Replication code: https://github.com/TigerHTaoLab/CASINO-parser

For manuscripts utilizing custom algorithms or software that are central to the research but not yet described in published literature, software must be made available to editors and reviewers. We strongly encourage code deposition in a community repository (e.g. GitHub). See the Nature Portfolio guidelines for submitting code & software for further information.

Data

Policy information about availability of data

All manuscripts must include a data availability statement. This statement should provide the following information, where applicable:

- Accession codes, unique identifiers, or web links for publicly available datasets
- A description of any restrictions on data availability
- For clinical datasets or third party data, please ensure that the statement adheres to our policy

All data needed to evaluate the conclusions in the paper are present in the paper and the Supplementary information. Additional data related to this paper may be requested from the authors. The computational data have been deposited in Zenodo at https://zenodo.org/record/5112635.

Field-specific reporting

Please select the one below that is the best fit for your research. If you are not sure, read the appropriate sections before making your selection.

☒ Life sciences ☐ Behavioural & social sciences ☐ Ecological, evolutionary & environmental sciences

For a reference copy of the document with all sections, see nature.com/documents/nr-reporting-summary-flat.pdf

Life sciences study design

All studies must disclose on these points even when the disclosure is negative.

Sample size	We generally used sample sizes of 3 biological replicates, which can provide necessary statistical support.
Data exclusions	Data were not excluded from analysis.
Replication	All biological experiments were successfully reproduced in independent experiments.
Randomization	The clones for silk protein production were picked up randomly during the experiments.
Blinding	No blinding was conducted, because blinding would not provide any reliable datasets for our all biochemical and genetic experiments.

Reporting for specific materials, systems and methods

We require information from authors about some types of materials, experimental systems and methods used in many studies. Here, indicate whether each material, system or method listed is relevant to your study. If you are not sure if a list item applies to your research, read the appropriate section before selecting a response.

Materials & experimental systems

n/a	Involved in the study	
☐	☒	Antibodies
☐	☒	Eukaryotic cell lines
☒	☐	Palaeontology and archaeology
☐	☒	Animals and other organisms
☒	☐	Human research participants
☒	☐	Clinical data
☒	☐	Dual use research of concern

Methods

n/a	Involved in the study	
☒	☐	ChIP-seq
☒	☐	Flow cytometry
☒	☐	MRI-based neuroimaging

Antibodies

Antibodies used	Anti-Nestin, ab105389, Rabbit monoclonal<br>Anti-GFAP, ab68428, Rabbit monoclonal<br>Goat polyclonal Secondary Antibody to Rabbit IgG - H&L (Alexa Fluor®488), ab150077
Validation	The validation statements are described on the manufacturer's website: https://www.abcam.com/primary-antibodies/how-we-validate-our-antibodies.

Eukaryotic cell lines

Policy information about cell lines

Cell line source(s)	Human GBM cell lines U251 were purchased from the Chinese Academy of Sciences (Shanghai, China).
Authentication	Short tandem repeat (STR) analysis for cell line authentication.
Mycoplasma contamination	Detecting mycoplasma contamination in cell cultures by polymerase chain reaction (MP0035 Sigma-Aldrich).
Commonly misidentified lines (See ICLAC register)	None.

Animals and other organisms

Policy information about studies involving animals; ARRIVE guidelines recommended for reporting animal research

Laboratory animals	The study did not involve laboratory animals. Bombyx mori cocoons were purchased from Sericultural Research Institute, Chinese Academy of Agricultural Sciences (Zhenjiang, China).
Wild animals	The study did not involve wild animals.
Field-collected samples	The study did not involve samples collected from the field.
Ethics oversight	No ehical approval or guidance was required because this study did not involve animals.

Note that full information on the approval of the study protocol must also be provided in the manuscript.
